# Multi-UAV Redeployment Optimization Based on Multi-Agent Deep Reinforcement Learning Oriented to Swarm Performance Restoration

**DOI:** 10.3390/s23239484

**Published:** 2023-11-28

**Authors:** Qilong Wu, Zitao Geng, Yi Ren, Qiang Feng, Jilong Zhong

**Affiliations:** 1 School of Reliability and Systems Engineering, Beihang University, Beijing 100191, China; wql_rse@buaa.edu.cn (Q.W.); gengzitao@buaa.edu.cn (Z.G.); renyi@buaa.edu.cn (Y.R.); 2 Defense Innovation Institute, Academy of Military Science, Beijing 100071, China; z_jilong@sina.cn

**Keywords:** distributed reconfiguration strategy, multi-agent deep reinforcement learning, unmanned aerial vehicle (UAV), UAV swarm redeployment

## Abstract

Distributed artificial intelligence is increasingly being applied to multiple unmanned aerial vehicles (multi-UAVs). This poses challenges to the distributed reconfiguration (DR) required for the optimal redeployment of multi-UAVs in the event of vehicle destruction. This paper presents a multi-agent deep reinforcement learning-based DR strategy (DRS) that optimizes the multi-UAV group redeployment in terms of swarm performance. To generate a two-layer DRS between multiple groups and a single group, a multi-agent deep reinforcement learning framework is developed in which a QMIX network determines the swarm redeployment, and each deep Q-network determines the single-group redeployment. The proposed method is simulated using Python and a case study demonstrates its effectiveness as a high-quality DRS for large-scale scenarios.

## 1. Introduction

Recently, mission planning associated with unmanned aerial vehicles (UAVs) has received considerable attention [1,2], and distributed artificial intelligence (AI) technologies have been extensively applied in multiple-UAV (multi-UAV) mission planning, enabling efficient decision-making and yielding high-quality solutions [3,4]. For missions in geographically decentralized environments, the focus is on deploying UAVs to their destinations and repositioning them to adapt to changing circumstances [5]. To minimize the costs of positioning UAVs, Masroor et al. [6] proposed a branch-and-bound algorithm that determines the optimal UAV deployment solution in emergency situations. Savkin et al. [7] employed a range-based reactive algorithm for autonomous UAV deployment. Nevertheless, many existing distributed algorithms lack the security necessary to achieve the global objective.

For the UAVs in a swarm, the placement of individual is important, but the completion of the swarm mission is the ultimate goal. Wang et al. [8] proposed a K-means clustering-based UAV deployment scheme that significantly improves the spectrum efficiency and energy efficiency of cellular uplinks at limited cost, while Yu et al. [9] introduced an evolutionary game-based adaptive dynamic reconfiguration mechanism that provides decision support for the cooperative mode design of unmanned swarm operations. These algorithms take static multi-swarm problems into account. However, some of the UAVs may suffer destruction or break down during a mission [10]. To deal with situations in which the swarm suffers unexpected destruction, adaptive swarm reconfiguration strategies are required [11].

Learning-based methods are gaining increasing attention for their flexibility and efficiency [12,13]. Deep reinforcement learning (DRL) has shown promising results in resolving the task assignment problems associated with multi-UAV swarms [14]. Samir et al. [15] combined DRL with joint optimization to achieve improved learning efficiency, although changes to the dynamic environment can hinder the implementation of this strategy. Zhang et al. [16] investigated a double deep Q-network (DQN) framework for long period UAV swarm collaborative tasks and designed a guided reward function to solve the convergence problem caused by the sparse returns of long period tasks. Huda et al. [17] investigated a surveillance application scenario using a hierarchical UAV swarm. In this case, they used a DQN to minimize the weighted sum cost. As a result, their DRL method exhibited better convergence and effectiveness than traditional methods. Zhang et al. [18] designed a DRL-based algorithm to find the optimal attack sequence for large-scale UAV swarm so that the purpose of destroying the target communication system can be achieved. Mou et al. [19] built a geometric way to project the 3D terrain surface into many weighted 2D patches and proposed a swarm DQN reinforcement learning algorithm to select patches for leader UAVs, which could cover the object area with little redundancies. Liu et al. [20] focused on a latency minimization problem for both communication and computation in a maritime UAV swarm mobile edge computing network, then they proposed a DQN and a deep deterministic policy gradient algorithm to optimize the trajectory of multi-UAVs and configuration of virtual machines. However, multi-agent DRL (MADRL) captures real-world situations more easily than DRL [21,22]. Hence, MADRL is considered an important topic of research. Xia et al. [22] proposed an end-to-end cooperative multi-agent reinforcement learning scheme that enables the UAV swarm to make decisions on the basis of the past and current states of the target. Lv et al. [23] proposed a MADRL-based UAV swarm communication scheme to optimize the relay selection and power allocation, then they designed a DRL-based scheme to improve the anti-jamming performance. Xiang et al. [24] established an intelligent UAV swarm model based on a multi-agent deep deterministic policy gradient algorithm, significantly improving the success rate of the UAV swarm in confrontations.

In summary, developments in distributed AI mean that swarm intelligence is now of vital strategic importance. Under this background, it is vital to develop multi-agent algorithms. However, few reconfiguration studies have investigated this distributed multi-agent scenario. Therefore, this paper proposes a MADRL-based distributed reconfiguration strategy (DRS) for the problem of UAV swarm reconfiguration after large-scale destruction. The main contributions of this paper are as follows: 

(1) UAV swarm reconfiguration is formulated so as to generate a swarm DRS considering detection missions and destruction. The finite number of UAVs is the constraint, and the coverage area forms the objective.

(2) MADRL-based swarm reconfiguration employs multi-agent deep learning and the Q-MIX network. Each agent, representing a group, uses reinforcement learning to select the optimal distributed reconfiguration (DR) actions. The Q-MIX network is used to synthesize the actions of each agent and output the final strategy.

(3) When the network has been trained well, the algorithm can effectively utilize various UAV swarm information to support DR decision-making. This enables efficient and steady multi-group swarm DR to achieve the mission objective.

The remainder of this paper is organized as follows. Section 2 presents the swarm mission framework. Section 3 elucidates the DRS, before Section 4 introduces a UAV swarm reconfiguration case study of detection missions. Finally, Section 5 presents the concluding remarks.

## 2. Problem Formulation

### 2.1. Mission, Destruction, and Reconfiguration

#### 2.1.1. Mission

**Figure 1:**
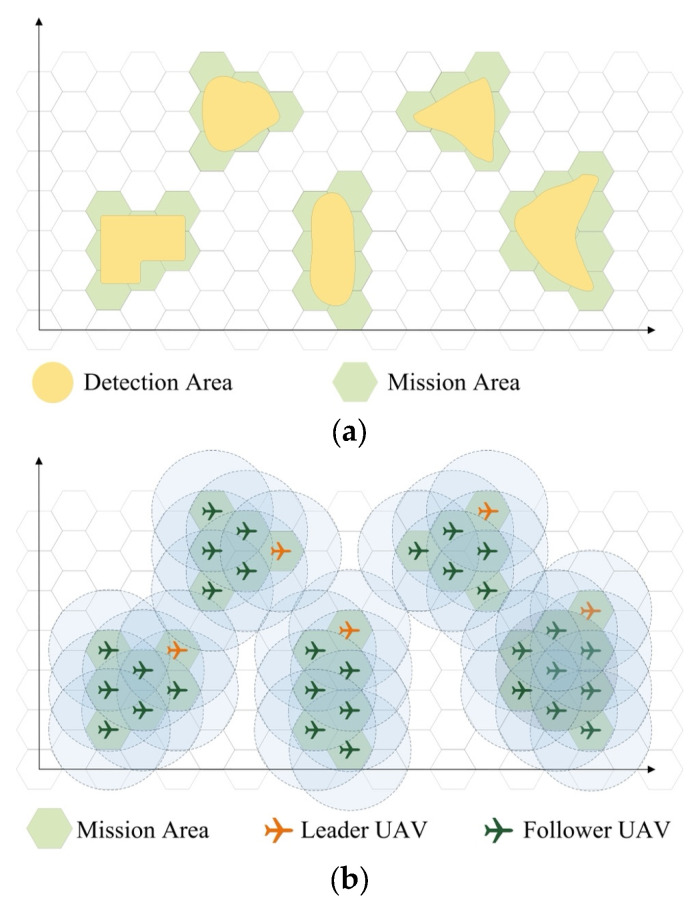
Mission and UAV swarm. (**a**) Detection and mission areas. (**b**) UAV swarm detection.

A detection mission containing *M* irregular detection areas is considered. As shown in Figure 1a, the detection areas (colored yellow) are divided into hexagons, which are inscribed hexagons of the mission areas (colored green).

The swarm detection mission area set can then be expressed as follows:

(1) $$D=\{{MA}_{1},\;{MA}_{2},\ldots ,{MA}_{m},\ldots ,{MA}_{M}\}$$

where each group mission area ${MA}_{m}$, m∈{1, 2, …, M}, is covered by a certain number of hexagons, as follows:

(2) $${MA}_{m}=\{{ma}_{m1},\;{ma}_{m2},\ldots ,{ma}_{mn},\ldots ,{ma}_{{mN}_{m}}\}$$

where $N_{m}$ is the total number of hexagons in group mission area ${\mathrm{MA}}_{m}$, and each hexagon represents a single UAV mission area ${ma}_{mn}$, m∈{1,2,…,M}, $n\in \{1,\;2,\;\ldots ,N_{m}\}$.

A UAV swarm, the size of which is determined by the detection area, is dispatched for a detection mission. Each area requires a group to execute the detection mission, and the number of UAVs in the group depends on the number of hexagons in the mission area. Furthermore, each group is formed of one leader UAV and several follower UAVs. To execute a detection mission, as shown in Figure 1b, the radius *R* of the UAV detection area is determined by the detection equipment installed on the UAVs.

The UAV swarm can then be expressed as follows:

(3) $$Swarm=\{G_{1},\;G_{2},\ldots ,G_{m},\ldots ,G_{M}\}$$

where each group $G_{m}$, m∈{1, 2, …, M}, performs detection in the group mission area ${\mathrm{MA}}_{m}$, as follows:

(4) $$G_{m}=\{U_{m1},\;U_{m2},\ldots ,U_{mn},\ldots ,U_{{mN}_{m}}\}$$

where $U_{mn}$ is the *m*-th UAV in group *n* and performs detection in UAV mission area ${ma}_{mn}$, m∈{1,2,…,M}, $n\in \{1,\;2,\;\ldots ,N_{m}\}$. The first UAV in each group is the leader of that group.

#### 2.1.2. Destruction

The UAV swarm may be subject to local and random destruction, and some UAVs may be destroyed. The effects of this destruction are used as inputs. Each UAV has two states: normal working and complete failure. When a UAV suffers destruction, it enters the failure state. When a leader UAV is destroyed, a follower UAV in the same group assumes the role of the leader of that particular group.

**Figure 2:**
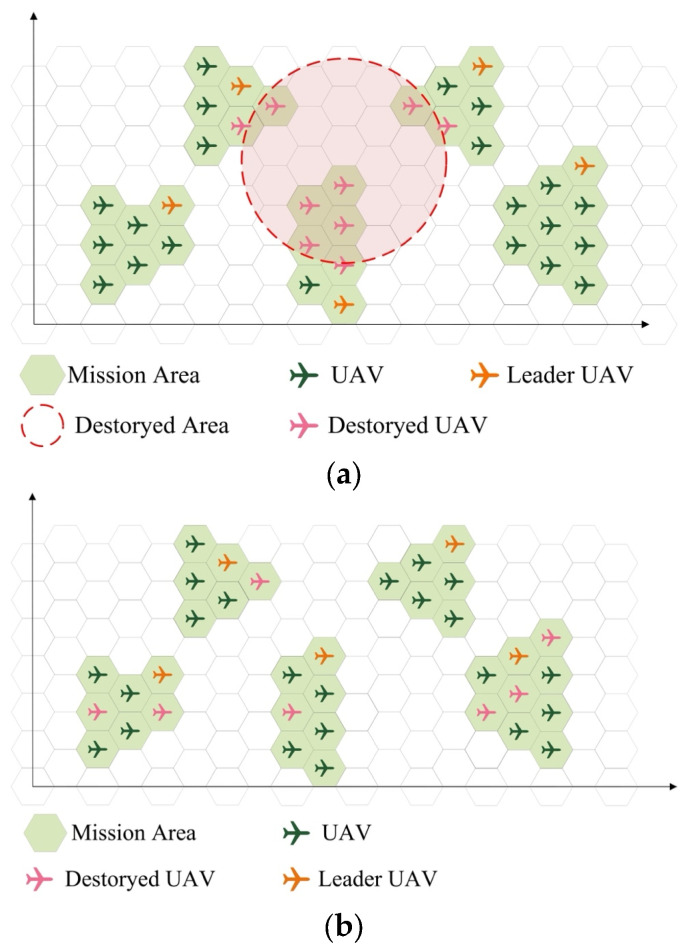
Different destruction types. (**a**) Local destruction. (**b**) Random destruction.

The scope of local destruction is represented by a circle with center coordinates $\left(i_{d},j_{d}\right)$ and radius $r_{d}$, as illustrated in Figure 2a. The values of $\left(i_{d},j_{d}\right)$ and $r_{d}$ are randomly generated.

Random destruction is characterized by a destruction scale, denoted as $S_{rand}$, which is also generated randomly. When random destruction occurs, $S_{rand}$ random UAVs transition from the normal state to the faulty state, as depicted in Figure 2b.

#### 2.1.3. Reconfiguration

UAV swarm reconfiguration is an autonomous behavior that adapts to changes in the environment to enable the execution of the task. When the swarm is affected by dynamic changes during task execution, the system can use a DRS to achieve global mission performance recovery and reconfiguration, thus ensuring mission continuity.

**Figure 3:**
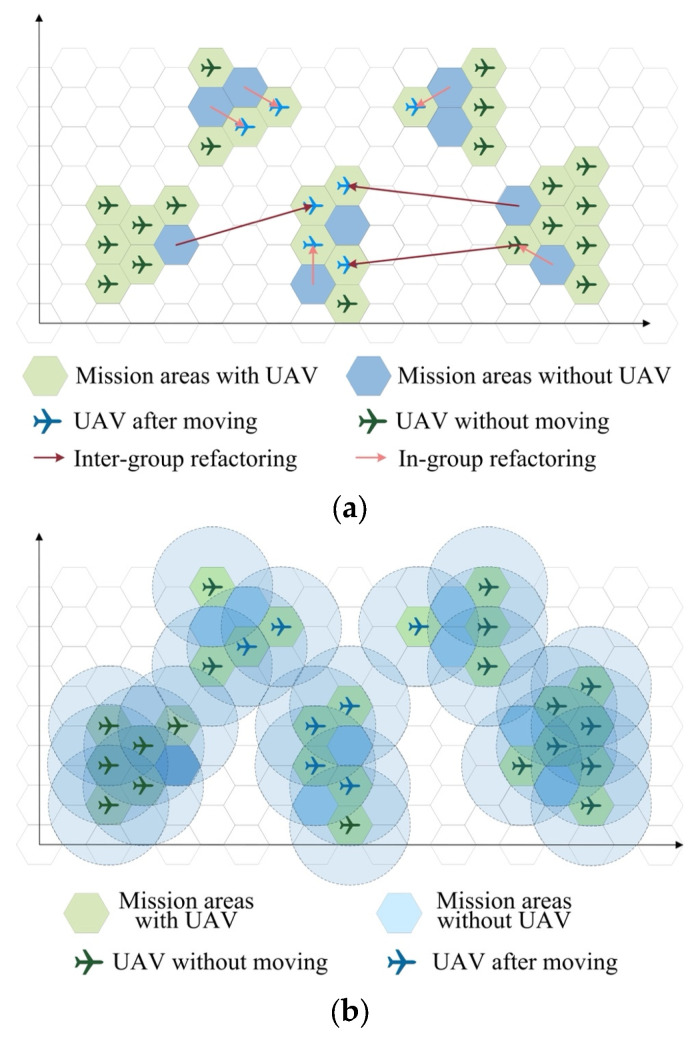
Reconfiguration progress. (**a**) Reconfiguration. (**b**) Detection.

When destruction occurs, the state of the UAV swarm is input into the reconstruction algorithm. The resulting strategy is communicated back to each UAV group. In-group reconstruction and inter-group reconstruction are applied to certain UAVs, as shown in Figure 3a. After the reconstruction is completed, all mission areas should be covered by the detection range of the UAVs, as shown in Figure 3b.

### 2.2. Objective, Constraints, and Variables

Over a finite time $\tau_{thr}$, swarm reconfiguration aims to maximize the total coverage area (TCA) $\varepsilon_{tot}$, which is the mission area detected by the UAVs. This can be expressed as follows:

(5) $$\varepsilon_{tot}(\tau )=\sum_{m=1}^{M}\sum_{n=1}^{N_{m}}\varepsilon_{mn}$$

where $\varepsilon_{tot}(\tau )$ is the TCA at the current time τ, $\varepsilon_{mn}$ is the detected area of mission area ${ma}_{mn}$; if ${ma}_{mn}$ is not covered, $\varepsilon_{mn}=0$.

The problem should be solved at the swarm level. Considering the number of remaining UAVs, the number of UAVs to be repositioned should be less than the number of normal working UAVs. Furthermore, the minimum area detected by the UAVs in each mission area must be set. Therefore, the reconfiguration problem can be expressed as follows:

(6) $$Max\;\varepsilon_{tot}\;s.t.\;\varepsilon_{m}\geq \varepsilon_{min}^{m}\;N_{move}^{m}\leq N_{normal}^{m}\;d\geq d_{min}\;m\in \{1,2,\ldots ,M\},n\in \{1,2,\ldots ,\;N_{m}\}$$

where $\varepsilon_{m}$ is the coverage area of group $G_{m}$, $\varepsilon_{min}^{m}$ is the specified minimum coverage area for group $G_{m}$, $N_{move}^{m}$ is the number of UAVs in $G_{m}$ that can be repositioned, $N_{normal}^{m}$ is the number of normal-working UAVs in $G_{m}$, *d* is the distance between two normal-working UAVs, and $d_{min}$ is the minimum allowable distance, which is the safety distance between UAVs. This problem considers the UAVs within the communication range. If a UAV exceeds the communication distance, it enters the faulty state due to communication failure.

The initial deployment status depends on whether there is a normal-working UAV at a certain hexagon for each group mission area ${MA}_{m}$. Then, the UAV swarm distribution deployment status can be represented by a I×J matrix *S*. The matrix element $s_{ij}$ = 1 if there is a normal-working UAV $U_{mn}$ in hexagon $H_{ij}$ and $s_{ij}$ = 0 if not. Therefore, the deployment status information of the UAV swarm can be expressed as follows:

(7) $$S=\left[\begin{array}{cccc} s_{11} & s_{12} & \cdots & s_{1J}\\ s_{21} & s_{22} & \ldots & s_{2J}\\ \vdots & \vdots & \ddots & \vdots \\ s_{\left(I-1\right)1} & s_{\left(I-1\right)2} & \cdots & s_{(I-1)J}\\ s_{I1} & s_{I2} & \cdots & s_{IJ} \end{array}\right]$$

## 3. MADRL-Based DR Method

**Figure 4:**
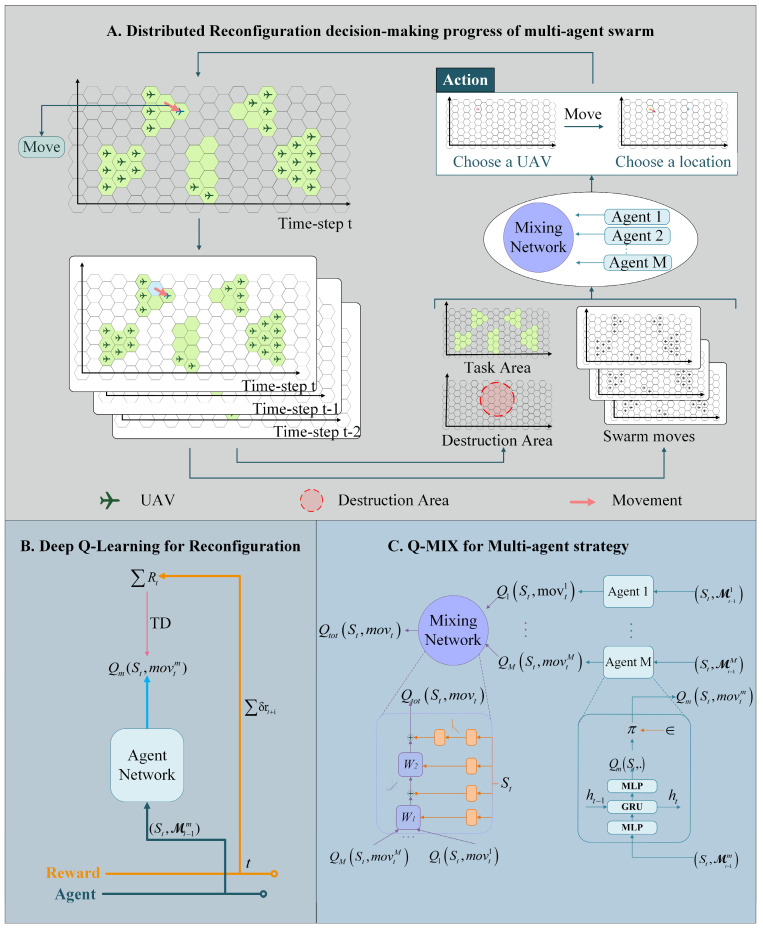
Multi-agent deep reinforcement learning framework.

An MADRL framework was developed to solve the DR problem described in the previous section, as shown in Figure 4. The framework consists of three parts: a reconfiguration decision-making progress, agent decision-making, and a neural network. The three parts of the framework are described in this section, and the reconfiguration decision-making process is illustrated in Figure 4A.

### 3.1. Reconfiguration Decision Process

The group agents choose the DRS for the UAV groups. The UAV swarm’s status matrix $S_{t}$ is used by the group agents as the main input. This process can be expressed as follows:

(8) $$f_{agent-m}(S_{t},M_{t-1}^{m})={mov}_{t}^{m}|[S_{t},M_{t-1}^{m}]$$

where $S_{t}$ represents the current state matrix of time step *t*, and $M_{t-1}^{m}$ represents the movement feature set of agent *m* which consists of this agent’s history of movement features $\left\{{mov}_{t-1}^{m},{mov}_{t-2}^{m},{mov}_{t-3}^{m}\right\}$. History of movement features are necessary, because the agents are not fully observable solely from the current state, since the DR decision-making is a sequence decision process. The movement feature of agent *m* can be described as ${mov}_{t}^{m}=[{loc}_{t}^{init},{loc}_{t}^{final}]$, where both ${loc}_{t}^{init}$ and ${loc}_{t}^{final}$ are the location matrices to describe the hexagons in the figures of Section 2. Each element in the location matric relates to a hexagon, and if the element is 1, the related hexagon is the chosen location. Both ${loc}_{t}^{init}$ and ${loc}_{t}^{final}$ have one element which is equal to 1, and the other elements are equal to 0. The location matrix ${loc}_{t}^{init}$ represents the initial location of the movement feature at time step *t*, while the location matrix ${loc}_{t}^{final}$ represents the final location of the same movement feature. Furthermore, if t<1, then both ${loc}_{t}^{init}$ and ${loc}_{t}^{final}$ are zero matrices. The output ${mov}_{t}^{m}$ represents the movement feature selected by the agent *m* at time step t. A swarm agent uses a QMIX network to combine the outputs of all group agents and choose the most efficient one. This can be expressed as follows:

(9) $$f_{agent-qmix}({mov}_{t}^{m}{|}_{m=1}^{m=M};S_{t},M_{t-1})={mov}_{t}|[S_{t},M_{t-1}]$$

where ${mov}_{t}^{m}{|}_{m=1}^{m=M}=\{{mov}_{t}^{1},{mov}_{t}^{2},\ldots ,{mov}_{t}^{M}\}$ represents the movement set of all group agents at time step *t*, $M_{t-1}$ represents the last swarm movement feature set which consists of this swarm’s history of movement features $\left\{{mov}_{t-1},{mov}_{t-2},{mov}_{t-3}\right\}$, and the output ${mov}_{t}|[S_{t},M_{t}]$ represents the final chosen movement feature for the swarm.

The DR process consists of mission and destruction features, DR action generation, and renewal features. These three components are described in the following subsections.

#### 3.1.1. Mission and Destruction Features

The destruction is randomly initialized at time $t_{d}$, and the status matrix *S* is then generated. The coverage area at this time is $\varepsilon_{A}(t_{d})=\varepsilon_{d}$.

To reconfigure the swarm and reach the maximum coverage rate, *M* agents, representing *M* different UAV groups, execute a sequence of DR actions. The DR action set is described as follows:

(10) $$\Phi =\{{act}_{t|mn}\}|t=1\;to\;T,\;m\in \left\{1,2,\ldots ,M\right\},n\in \{1,2,\ldots ,N_{m}\}$$

where the ${act}_{t|mn}$ is the DR action of ${UAV}_{mn}$ at time step *t*. This DR action is defined as ${act}_{t|mn}=[cen(H_{ij}),cen(H_{i”j”})]$ which means that the ${UAV}_{mn}$ in hexagon $H_{ij}$ moves to the target hexagon $H_{i”j”}$. The parameter $cen(H_{ij})$ represents the center location of hexagon $H_{ij}$, and the action ${act}_{t|mn}$ is generated according to the movement feature ${mov}_{t}$. The DR action set of group *m* can be described as follows:

(11) $$\Phi_{m}=\{{act}_{t|mn}\}|n=1\;to\;N_{m},\;m\in \left\{1,2,\ldots ,M\right\},t\in \{1,2,\ldots ,T\}$$

After the DR action has finished, agent *m* uses a search algorithm to select the next DR action ${act}_{mn|t}$ for ${UAV}_{mn}$ in group $G_{m}$, or chooses to finish the reconfiguration process. This process is repeated in each time step *t*. The neural network of agent *m* (see Section 3.2) can be described as follows:

(12) $$Q_{m}(S_{t},{mov}_{t}^{m})=f_{DQN-m}(S_{t},M_{t}^{m})$$

where $Q_{m}(S_{t},{mov}_{t}^{m})$ is the value of the movement feature ${mov}_{t}^{m}$ at time step *t*.

Each time step corresponds to a realistic period of time, the length of which is proportional to the distance the UAV moves in this time step.

#### 3.1.2. Reconfiguration Action Generation

For the DR action ${act}_{t|mn}$, once complete, the moving UAV is considered to perform the detection mission at the new location, then the status matrix $S_{t}$ can be updated. The term $\varepsilon_{A}(t)$ can be calculated according to (5) after the movement. The objective of agent *m* is to achieve the maximum coverage area as efficiently as possible. Thus, the reward should include both the coverage area and reconfiguration time. All agents use the same reward function, and the reward at time step t is defined as follows:

(13) $$R_{t}=\sum_{\zeta =0}^{T-t}\delta^{\zeta }\int_{\tau_{t+\zeta -1}}^{\tau_{t+\zeta }}\left(1-\frac{\varepsilon_{tot}(\tau )}{\varepsilon_{0}}\right)d\tau$$

where $R_{t}$ is the reward at time step t, $\tau_{t+\zeta }$ is the reconfiguration time of time step (t+ζ), $\tau_{t+\zeta -1}$ is the reconfiguration time of time step (t+ζ−1), $\varepsilon_{0}$ is the initial TCA, δ is the discounted factor, and $\tau_{T}$ is the time to finish reconfiguration (TTFR).

For agent *m*, an optimization algorithm is used to select the best movement feature of UAV group *m*. For each time step *t*, the DQN of agent *m* outputs a movement value quantity $Q_{m}(S_{t},{mov}_{t}^{m})$, then this agent outputs a movement feature ${mov}_{t}^{m}$. A QMIX network is used to select the most effective action from all possible actions.

The mixing network has two parts: a parameter generating network and an inference network. The former receives the global state $S_{t}$ and generates the neuron weights and deviations. The latter receives the control quantity $Q_{m}(S_{t},{mov}_{t}^{m})$ from each agent and generates the global utility value $Q_{tot}$ with the help of the neuron weights and deviations.

The movement utility value $Q_{tot}$ is used to formulate the final decision for the whole swarm (see Section 3.3), as expressed in (15).

#### 3.1.3. Renewal Features

Once the swarm has finished ${act}_{t|mn}$, the state matrix and feature set $\left[S_{t},A_{t}\right]$ is used as the new input to the algorithm. The algorithm continues to run and outputs new movement actions or takes the decision to end the reconfiguration process.

### 3.2. Deep Q-Learning for Reconfiguration

The agents use the deep Q-learning algorithm to evaluate the movement action, in which the action-value function is represented by a deep neural network parameterized by ϑ. The movement feature ${mov}_{t}^{m}$ has a movement value function of $Q_{m}(S_{t},{mov}_{t}^{m})=E_{S_{t+1;\infty },{\;mov}_{t+1;\infty }^{m}}[\sum R_{t}|S_{t},{mov}_{t}^{m}]$, where $\sum R_{t}=\sum_{i=0}^{\infty }\delta^{i}r_{t+i}$ is the discounted return and δ is the discount factor.

The transition tuple of each movement action of the group agent *m* is stored as $\left[S,{mov}^{m},R,S^{\prime }\right]$, where S is the state before mov, mov is the selected mobile movement feature, R is the reward for this movement, and $S^{\prime }$ is the state after the movement has finished. ϑ is learned by sampling batches of *b* transitions and minimizing the squared temporal-difference error:

(14) $$L\left(\vartheta \right)=\sum_{i=1}^{b}\left[{\left(\gamma_{i}^{DQN}-Q_{m}(S,{mov}^{m};\vartheta )\right)}^{2}\right]$$

where $\gamma^{DQN}=R+\delta {max}_{{mov}^{\prime }}Q_{m}(S^{\prime },{mov}^{m^{\prime }};\vartheta^{-})$, $\vartheta^{-}$ represents parameters of the target network that are periodically copied from ϑ and held constant for several iterations, *b* is the batch size of transitions sampled from the replay buffer, $Q_{m}(S,{mov}^{m};\vartheta )$ is the utility value of ${mov}^{m}$.

### 3.3. QMIX for Multi-Agent Strategy

The QMIX network is applied to the generated swarm-level DR action. The network represents $Q_{tot}$ as a monotone function for mixing the individual value functions $Q\left(S_{t},{mov}_{t}\right)$ of each agent. This can be expressed as follows:

(15) $$Q_{tot}\left(S,mov\right)=f_{qmix}\left(Q_{m}\left(S,{mov}^{m}\right){|}_{m=1}^{m=M}\right)$$

where $Q_{m}\left(S,{mov}^{m}\right){|}_{m=1}^{m=M}$ is the movement value set, and the $Q_{tot}\left(S,mov\right)$ is a joint movement value of the swarm. The monotonicity of (15) can be enforced by the partial derivative relation $\frac{\partial Q_{tot}}{\partial Q_{m}}\geq 0,\;m\in [1,M]$. To ensure this relationship, QMIX consists of agent networks, a mixing network, and a set of hypernetworks, as shown in Figure 4C.

For each agent *m*, there is one agent network representing the individual value function $Q_{m}\left(S,{mov}^{m}\right)$. The agent networks are represented as deep recurrent Q-networks (DRQNs). At each time step, the DRQNs receive the status $S_{t}$ and last movement ${mov}_{t}$ as input and output a value function $Q_{m}\left(S,{mov}^{m}\right)$ to the mixing network.

The mixing network is a feedforward neural network that monotonically mixes all $Q_{m}\left(S,{mov}^{m}\right)$ with nonnegative weights. The weights of the mixing network are generated by separate hypernetworks, each of which generates the weight of one layer using the status $S_{t}$. The biases of the mixing network are produced in the same manner but are not necessarily nonnegative. The final bias is produced by a two-layer hypernetwork.

The whole QMIX network is trained end-to-end to minimize the following loss:

(16) $$L\left(\vartheta \right)=\sum_{i=1}^{b}\left[{\left(\gamma_{i}^{DQN}-Q_{tot}(S,mov;\vartheta )\right)}^{2}\right]$$

where $\gamma^{DQN}=R+\delta {max}_{{mov}^{\prime }}Q_{tot}(S^{\prime },{mov}^{\prime };\vartheta^{-})$, $Q_{tot}(S,mov;\vartheta )$ is the globe utility value of mov.

## 4. Case Study

A case study of UAV swarm reconfiguration was simulated using Python. The numerical simulation is described from the perspective of optimal UAV swarm reconfiguration. The effectiveness of the proposed DR decision-making method is validated using the reconfiguration results under different scenarios. In this section, a fixed-wing UAV swarm is considered, although the proposed method is also applicable to other types of UAV swarms.

### 4.1. UAV Swarm Reconfiguration

#### 4.1.1. Mission

**Figure 5:**
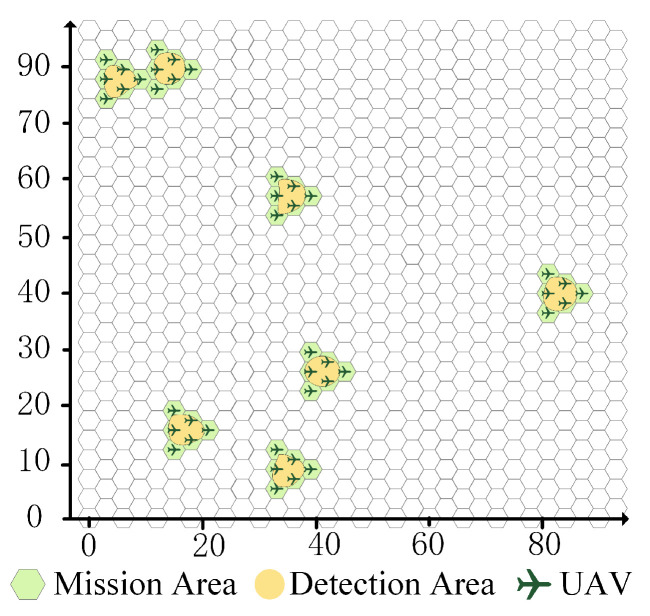
Mission and UAV swarm deployment.

A detection mission containing seven irregular detection areas is randomly generated, as shown in Figure 5. The yellow areas represent the detection areas, and the map is divided into hexagons. The hexagons of the mission areas (colored green) need to cover the detection areas. A UAV swarm with 7 × 6 UAVs is simulated to execute this detection mission; the initial location information of each UAV is presented in Table 1.

**Figure 6:**
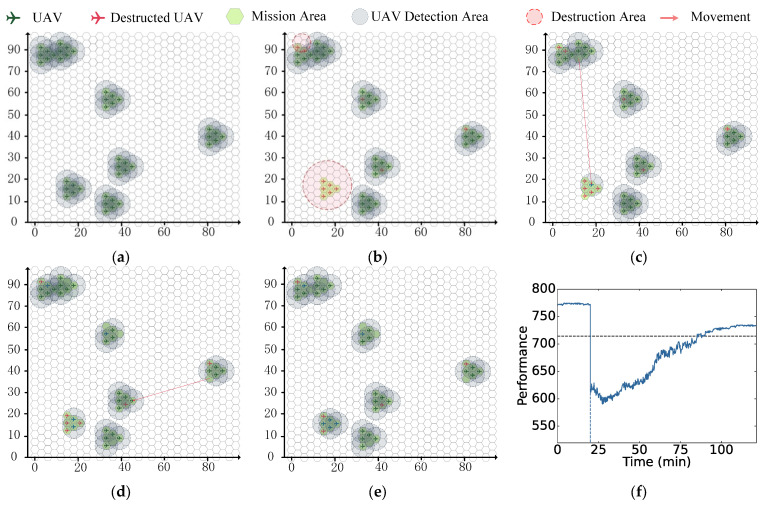
Destruction and reconfiguration of UAVs. (**a**) Initial state. (**b**) Destruction. (**c**) Reconfiguration step 1. (**d**) Reconfiguration step T. (**e**) Redeployment. (**f**) Performance.

From the UAV swarm deployment in Figure 5, the initial detection mission state is shown in Figure 6a, where each light-gray circle represents the detection area of one UAV. In this case, all UAVs in the swarm are assumed to have the same detection radius of $3\sqrt{3}$ km, and the initial TCA is $\varepsilon_{tot}$ = 770.59 km^2^. Furthermore, the safety distance is assumed to be 0.2 km. The detection radius and safety distance can also be assigned based on the actual regions.

#### 4.1.2. Destruction

The destruction states were randomly generated. Two kinds of destruction, namely local and random destruction, were considered simultaneously. For local destruction, the destruction center is a randomly sampled point on the mission area, and the destruction area is a randomly generated irregular polygon. For random destruction, the number of destroyed UAVs is assumed to follow the Poisson distribution with λ=1.

For the mission and swarm deployment case in Figure 6a, the generated destruction states are illustrated in Figure 6b, and consist of two local destruction areas and a random destruction with three UAVs. The destruction centers of these two local destruction areas are (18, 17.32) and (5, 83.13), and the radii are 11 and 4, respectively. The destroyed UAVs in Figure 6b can be described as {*U*_1,1_, *U*_1,5_, *U*_3,2_, *U*_4,3_, *U*_5,4_, *U*_6,1_, *U*_6,2_, *U*_6,3_, *U*_6,4_, *U*_6,5_, *U*_6,6_}, including both local destruction and random destruction. After this destruction process, the current total coverage area is $\varepsilon_{tot}$ = 615.02 km^2^. All of the destruction information is presented in Table 2.

#### 4.1.3. Reconfiguration

For the reconfiguration process, the initial time step and the final time step are shown in Figure 6c,d, respectively. The UAV colored yellow represents the initial location of this reconfiguration process, while the UAV colored blue represents the final location. The red arrow represents the reconfiguration route from the initial location to the final location, which can be generated by the movement feature set M according to (9). Each reconfiguration action is generated by an agent of the proposed multi-agent framework according to (9). For the case in Figure 6c,d, the DR action set Φ is listed in Table 3.

After this reconfiguration process, the UAV swarm has finished its redeployment. The current detection state is shown in Figure 6e. All UAVs in the swarm are assumed to have the same speed of 50 km/h. The speed can also be assigned based on the actual regions. The TCA is considered as a metric of UAV swarm performance. During this reconfiguration process, the UAV swarm performance exhibits a fluctuating upward trend, as shown in Figure 6f. The black dashed line in Figure 6f represents the TCA threshold $\varepsilon_{thr}$, which is assumed to be 714 km^2^. This TCA threshold can also be assigned on the basis of actual conditions. After finishing the reconfiguration process, the final TCA is $\varepsilon_{tot}(\tau_{T})$ = 732.31 km^2^.

### 4.2. Discussion

In addressing the UAV swarm reconfiguration, the main objective is to generate an optimal feasible strategy. Extended analyses are now presented covering the method performance and the influence of various factors.

#### 4.2.1. Different Algorithms

**Figure 7:**
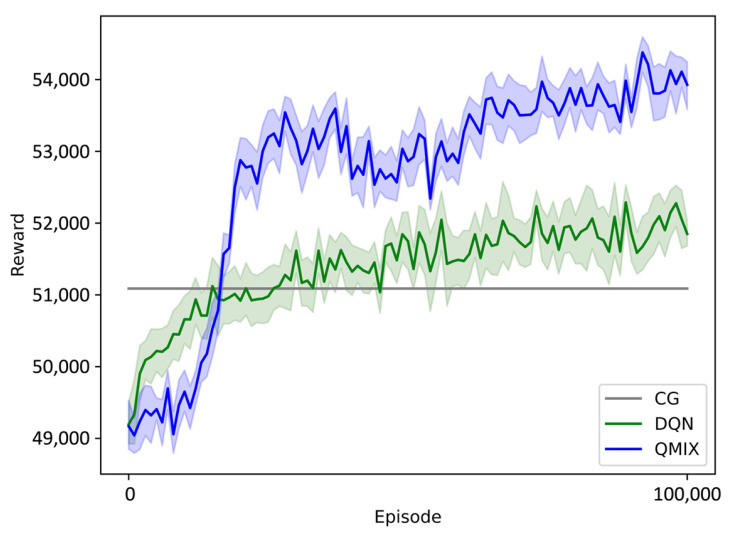
Reward of different algorithms.

This section evaluates the performance of the proposed QMIX method, although the DQN method and a cooperative game (CG) method [25] have also been used to generate this UAV swarm DRS. We used a single machine with one Intel i9 7980XE CPU and four RTX2080 TI-A11G GPUs to train the QMIX network and the DQN network. During the training process, each episode generated a DRS for the randomly generated mission and destruction, as described in Section 4.1. This section presents the results of the following assessment process: for each training procedure, the training is paused every 100 episodes and the method runs 10 independent episodes with greedy action selection. Figure 7 plots the mean reward across these 10 runs for each method with independent mission and destruction details. As the 10 independent episodes are fixed, the mean reward of the CG method is a constant value. Thus, the reward curve of CG method is a straight line. The shading around each reward curve represents the standard deviation across the 10 runs. Over the training process, 100,000 episodes were executed for each method. The reward curves of these two methods fluctuate upward. In the first 17,000 episodes, the DQN method exhibits faster growth than the QMIX method. However, QMIX achieves a higher upper bound of the reward curve after 20,000 episodes. QMIX is noticeably stronger in terms of the final DR decision-making performance. The superior representational capacity of QMIX combined with the state information provides a clear benefit over the DQN method.

#### 4.2.2. Different Destruction Cases

**Figure 8:**
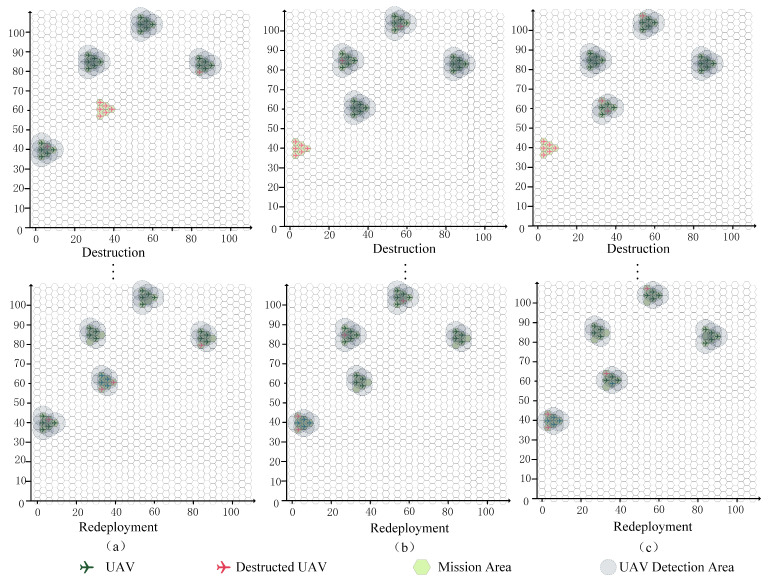
Reconfiguration under different destruction cases (**a**–**c**).

For a given mission and swarm scale, the destruction process was randomly generated. The redeployment results were obtained by executing the QMIX reconfiguration strategy, as shown in Figure 8. The three subgraphs demonstrate the initial deployment status, the destruction status, and the redeployment results of the proposed QMIX algorithm. The geographical distributions of all mission areas and the swarm with 5 × 6 UAVs are the same in the three subgraphs, while the destruction states are different. After the reconfiguration process, the redeployment results in the three subgraphs demonstrate that the proposed QMIX method exhibits stable performance for this reconfiguration decision-making problem with different destruction patterns. This is because, during the training process, UAV destruction is generated randomly.

**Figure 9:**
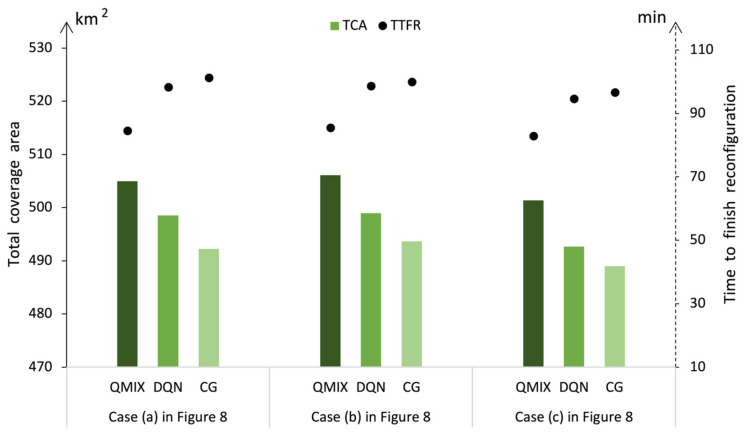
Reconfiguration under different destruction cases.

In addressing the UAV swarm redeployment, the main objective was to obtain an optimal feasible DRS strategy. Extended analyses of the optimization strategy were conducted to determine the influences of different methods. The QMIX method with high efficiency was proposed for this optimization strategy, while the DQN method and the CG method had also been used to solve the three destruction cases in Figure 8. The QMIX method gives optimal solutions with better TCA $\varepsilon_{tot}(\tau_{T})$ and less TTFR $\tau_{T}$ than the other methods, as shown in Figure 9. The proposed method achieves the better solution, since these two methods may lead to local optima, such as a situation in which multiple UAVs have to spend more time moving during the reconfiguration process. The efficiencies of the methods are analyzed in Table 4. According to these results, the solution speeds of the QMIX and DQN are close, while the solution speed of CG method is significantly slower than the other two methods.

#### 4.2.3. Different Swarm Scales

**Figure 10:**
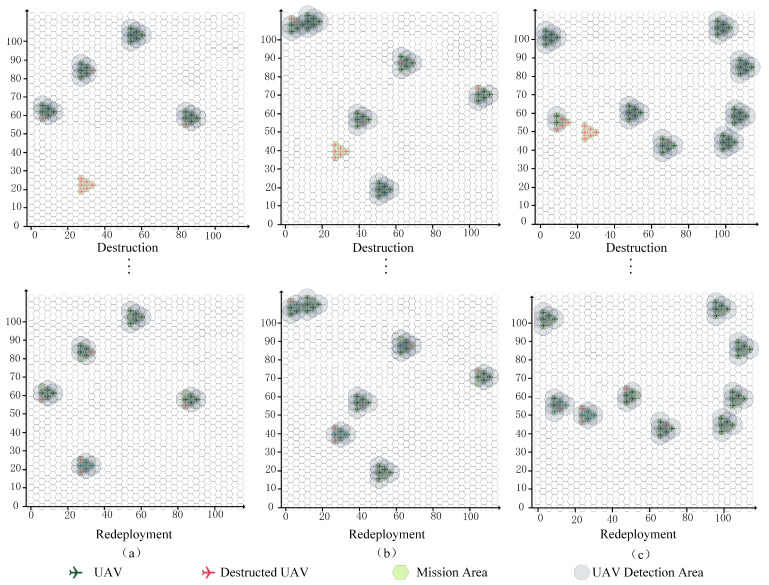
Reconfiguration under different swarm scales (**a**–**c**).

Under different missions and swarm scales, the redeployment results obtained by executing the QMIX reconfiguration strategy are shown in Figure 10. The three subgraphs demonstrate the different deployment missions, the destruction status, and the redeployment results of the proposed method. The geographical distributions of all mission areas were randomly generated in the three subgraphs, and the initial swarm scales were 5 × 6, 7 × 6, and 9 × 6. Then, the destruction states were randomly generated. After the reconfiguration process, the redeployment results in the three subgraphs demonstrate that the proposed QMIX method exhibits stable performance under the different missions and swarm scales. During the training process, the missions and swarm scales were generated randomly. Thus, the superior representational capacity of QMIX combined with the mission state and swarm state information provides a clear benefit in terms of reconfiguration decision-making performance.

**Figure 11:**
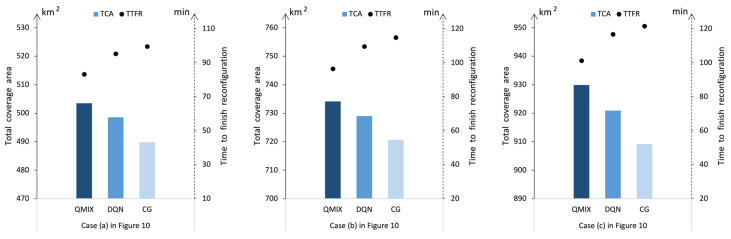
Reconfiguration under different swarm sizes.

Again, keeping the same cases as in Figure 10 and using the QMIX method, the DQN method, and the CG method, we also analyzed the differences in algorithm performance. Under different missions and swarm scales, the QMIX method also gives optimal solutions with better TCA $\varepsilon_{tot}(\tau_{T})$ and less TTFR $\tau_{T}$ than the other methods, as shown in Figure 11. The efficiencies of the methods are analyzed in Table 5. According to these results, the solution speeds of the QMIX and DQN are close for each case, while the solution speed of CG method is significantly slower than the other two methods. Furthermore, the solution speeds of the QMIX and DQN are stable, and they do not exponentially decrease as the swarm scales increase. However, the solution speed of the CG method clearly decreases as the swarm scale increases. Thus, these results show that the proposed QMIX method exhibits stable DR decision-making performance for swarms with different scales.

## 5. Conclusions

Distributed AI is gradually being applied to multi-UAVs. This paper has focused on DR decision-making for UAV swarm deployment optimization using a proposed MADRL framework. A two-layered decision-making framework based on MADRL enables UAV swarm redeployment, which maximizes swarm performance. Simulations using Python have demonstrated that the proposed QMIX method can generate a globally optimal DRS for UAV swarm redeployment. Furthermore, the results of the case study show that the QMIX method achieves a better swarm performance with less reconfiguration time than the other methods and exhibits stable and efficient solution speed. The DR decision-making problem considered in this paper is one of redeployment decision-making; the initial deployment planning was not addressed. Future research should emphasize the integration of UAV swarm initial deployments into decision-making frameworks.

## Figures and Tables

**Table 1:** Location of each UAV.

$G_{1}$	${UAV}_{1,1}$	${UAV}_{1,2}$	${UAV}_{1,3}$	${UAV}_{1,4}$	${UAV}_{1,5}$	${UAV}_{1,6}$
Location	(3, 74.48)	(3, 77.94)	(3, 81.41)	(6, 76.21)	(6, 79.67)	(9, 77.94)
$G_{2}$	${UAV}_{2,1}$	${UAV}_{2,2}$	${UAV}_{2,3}$	${UAV}_{2,4}$	${UAV}_{2,5}$	${UAV}_{2,6}$
Location	(12, 76.21)	(12, 79.67)	(12, 83.14)	(15, 77.94)	(15, 81.41)	(18, 79.67)
$G_{3}$	${UAV}_{3,1}$	${UAV}_{3,2}$	${UAV}_{3,3}$	${UAV}_{3,4}$	${UAV}_{3,5}$	${UAV}_{3,6}$
Location	(33, 53.69)	(33, 57.16)	(33, 60.62)	(36, 55.43)	(36, 58.89)	(39, 57.16)
……						
$G_{6}$	${UAV}_{6,1}$	${UAV}_{6,2}$	${UAV}_{6,3}$	${UAV}_{6,4}$	${UAV}_{6,5}$	${UAV}_{6,6}$
Location	(15, 12.12)	(15, 15.59)	(15, 19.05)	(18, 13.86)	(18, 17.32)	(21, 15.69)
$G_{7}$	${UAV}_{7,1}$	${UAV}_{7,2}$	${UAV}_{7,3}$	${UAV}_{7,4}$	${UAV}_{7,5}$	${UAV}_{7,6}$
Location	(33, 5.20)	(33, 12.12)	(33, 8.66)	(36, 6.93)	(36, 10.39)	(39, 8.66)

**Table 2:** Destruction information.

Destruction	Parameter	Parameter Value
Local destruction 1	Destruction center	(18, 17.32)
	Destruction radius	11
	Destroyed UAVs	${UAV}_{6,1}$, ${UAV}_{6,2}$, ${UAV}_{6,3}$, ${UAV}_{6,4}$, ${UAV}_{6,5}$, ${UAV}_{6,6}$
Local destruction 2	Destruction center	(5, 83.13)
	Destruction radius	4
	Destroyed UAVs	${UAV}_{1,1}$, ${UAV}_{1,5}$
Random destruction	Destroyed UAVs	${UAV}_{3,2}$, ${UAV}_{4,3}$, ${UAV}_{5,4}$

**Table 3:** Reconfiguration action set.

Swarm Scale	UAV	Agent	Reconfiguration Action	
			Initial Location	Final Location
7 × 6 UAVs	${UAV}_{1,6}$	agent 1	(9, 77.94)	(6, 79.67)
	${UAV}_{2,5}$	agent 2	(15, 81.41)	(15, 15.59)
	${UAV}_{3,3}$	agent 3	(33, 60.62)	(18, 17.32)
	${UAV}_{3,6}$	agent 3	(39, 57.16)	(33, 57.16)
	……			
	${UAV}_{4,1}$	agent 4	(82, 36.37)	(21, 15.59)
	${UAV}_{7,4}$	agent 7	(36, 6.93)	(18, 13.86)

**Table 4:** Running time (in seconds) of different methods under different destruction cases.

Different Destruction Cases	QMIX	DQN	CG
Case (a) in Figure 8	20.652	19.215	43.642
Case (b) in Figure 8	20.857	19.618	44.258
Case (c) in Figure 8	20.116	19.128	42.289

**Table 5:** Running time (in seconds) of different methods under different swarm sizes.

Different Swarm Cases	QMIX	DQN	CG
Case (a) in Figure 10	20.542	19.942	44.845
Case (b) in Figure 10	27.031	26.531	70.275
Case (c) in Figure 10	32.816	32.116	120.389

## Data Availability

Data are contained within the article.

## References

[1] Sun Z.C., Yen G.G., Wu J., Ren H., An H., Yang J. (2023). Mission planning for energy-efficient passive UAV radar imaging system based on substage division collaborative search. IEEE Trans. Cybern..

[2] Jinqiang H., Husheng W., Renjun Z., Rafik M., Xuanwu Z. (2021). Self-organized search-attack mission planning for UAV swarm based on wolf pack hunting behavior. J. Syst. Eng. Electron..

[3] Cheng N., Wu S., Wang X., Yin Z., Li C., Chen W., Chen F. (2023). AI for UAV-assisted IoT applications: A comprehensive review. IEEE Internet Things J..

[4] Khan M.A., Kumar N., Mohsan S.A.H., Khan W.U., Nasralla M.M., Alsharif M.H., Żywiołek J., Ullah I. (2023). Swarm of UAVs for network management in 6G: A technical review. IEEE Trans. Netw. Serv. Manag..

[5] Li X.W., Yao H.P., Wang J.J., Xu X., Jiang C., Hanzo L. (2019). A near-optimal UAV-aided radio coverage strategy for dense urban areas. IEEE Trans. Veh. Technol..

[6] Masroor R., Naeem M., Ejaz W. (2021). Efficient deployment of UAVs for disaster management: A multi-criterion optimization approach. Comput. Commun..

[7] Savkin A.V., Huang H.L. (2021). Range-based reactive deployment of autonomous drones for optimal coverage in disaster areas. IEEE Trans. Syst. Man Cybern. Syst..

[8] Wang J., Liu M., Sun J.L., Gui G., Gacanin H., Sari H., Adachi F. (2021). Multiple unmanned-aerial-vehicles deployment and user pairing for nonorthogonal multiple access schemes. IEEE Internet Things J..

[9] Yu M.G., Niu Y.J., Liu X.D., Zhang D.G., Peng Z., He M., Luo L. (2023). Adaptive dynamic reconfiguration mechanism of unmanned swarm topology based on an evolutionary game. J. Syst. Eng. Electron..

[10] Wang Y.Z., Yue Y.F., Shan M., He L., Wang D. (2021). Formation reconstruction and trajectory replanning for multi-UAV patrol. IEEE/ASME Trans. Mechatron..

[11] Bouhamed O., Ghazzai H., Besbes H., Massoud Y. (2020). A generic spatiotemporal scheduling for autonomous UAVs: A reinforcement learning-based approach. IEEE Open J. Veh. Technol..

[12] Zhang H., Li J., Qi Z., Aronsson A., Bosch J., Olsson H.H. Deep Reinforcement Learning for Multiple Agents in a Decentralized Architecture: A Case Study in the Telecommunication Domain. Proceedings of the IEEE 20th International Conference on Software Architecture Companion (ICSA-C).

[13] Ren L., Wang C., Yang Y., Cao Z. A Learning-Based Control Approach for Blind Quadrupedal Locomotion with Guided-DRL and Hierarchical-DRL. Proceedings of the IEEE International Conference on Robotics and Biomimetics (ROBIO).

[14] Xu J., Guo Q., Xiao L., Li Z., Zhang G. Autonomous Decision-Making Method for Combat Mission of UAV Based on Deep Reinforcement Learning, Electronic and Automation Control. Proceedings of the Conference (IAEAC).

[15] Samir M., Assi C., Sharafeddine S., Ebrahimi D., Ghrayeb A. (2020). Age of Information Aware Trajectory Planning of UAVs in Intelligent Transportation Systems: A Deep Learning Approach. IEEE Trans. Veh. Technol..

[16] Zhang Y., Li Y., Wu Z., Xu J. (2023). Deep reinforcement learning for UAV swarm rendezvous behavior. J. Syst. Eng. Electron..

[17] Huda S.M.A., Moh S. (2023). Deep reinforcement learning-based computation offloading in uav swarm-enabled edge computing for surveillance applications. IEEE Access.

[18] Zhang N., Liu C., Ba J. (2023). Decomposing FANET to Counter Massive UAV Swarm Based on Reinforcement Learning. IEEE Commun. Lett..

[19] Mou Z., Zhang Y., Gao F., Wang H., Zhang T., Han Z. (2021). Deep Reinforcement Learning Based Three-Dimensional Area Coverage With UAV Swarm. IEEE J. Sel. Areas Commun..

[20] Liu Y., Yan J., Zhao X. (2022). Deep Reinforcement Learning Based Latency Minimization for Mobile Edge Computing with Virtualization in Maritime UAV Communication Network. IEEE Trans. Veh. Technol..

[21] Zhang R., Zong Q., Zhang X., Dou L., Tian B. (2022). Game of drones: Multi-uav pursuit-evasion game with online motion planning by deep reinforcement learning. IEEE Trans. Neural Netw. Learn. Syst..

[22] Xia Z., Du J., Wang J., Jiang C., Ren Y., Li G., Han Z. (2022). Multi-agent reinforcement learning aided intelligent UAV swarm for target tracking. IEEE Trans. Veh. Technol..

[23] Lv Z., Xiao L., Du Y., Niu G., Xing C., Xu W. (2023). Multi-Agent Reinforcement Learning based UAV Swarm Communications against Jamming. IEEE Trans. Wirel. Commun..

[24] Xiang L., Xie T. Research on UAV Swarm Confrontation Task Based on MADDPG Algorithm. Proceedings of the 5th International Conference on Mechanical, Control and Computer Engineering (ICMCCE).

[25] Feng Q., Bi W., Chen Y., Ren Y., Yang D. (2017). Cooperative Game Approach based on Agent Learning for Fleet Maintenance Oriented to Mission Reliability. Comput. Ind. Eng..

